# Characteristics of pediatric adverse drug reaction reports in the Japanese Adverse Drug Event Report Database

**DOI:** 10.1186/s40360-020-00412-7

**Published:** 2020-05-24

**Authors:** Aoi Noda, Takamasa Sakai, Taku Obara, Makoto Miyazaki, Masami Tsuchiya, Gen Oyanagi, Yuriko Murai, Nariyasu Mano

**Affiliations:** 1 Division of Preventive Medicine and Epidemiology, Tohoku University Tohoku Medical Megabank Organization, Sendai, Miyagi Japan; 2 Tohoku University Graduate School of Medicine, Sendai, Miyagi Japan; 3 Department of Pharmaceutical Sciences, Tohoku University Hospital, Sendai, Miyagi Japan; 4 Drug Informatics, Faculty of Pharmacy, Meijo University, Nagoya, Aichi Japan; 5 Laboratory of Clinical Pharmacy, Tohoku University Graduate School of Pharmaceutical Sciences, Sendai, Miyagi Japan; 6 Department of Pharmacy, Miyagi Cancer Center, Natori, Miyagi Japan; 7 Department of Clinical Pharmaceutics, Tohoku Medical and Pharmaceutical University, Sendai, Miyagi Japan

**Keywords:** Adverse drug reaction, Pediatric patients, Children, The JADER, Spontaneous reports, Drug safety, Pharmacovigilance, Signal detection

## Abstract

**Background:**

There are no reports on investigations of the characteristics of adverse drug reaction (ADR) reports for pediatric patients in the Japanese Adverse Drug Event Report database (JADER) and the utility of database for drug safety surveillance in these patients.

**Method:**

We aimed to evaluate ADR reports for pediatric patients in the JADER. We used spontaneous ADR reports included in the JADER since April 1, 2004, to December 31, 2017, which was downloaded in April 2018. In a total of 504,407 ADR reports, the number of spontaneous reports was 386,400 (76.6%), in which 37,534 (7.4%) were unknown age reports. After extraction of 27,800 ADR reports for children aged < 10 and 10–19 years, we excepted for ADR reports associated with a vaccine (*n* = 6355) and no-suspected drug reports (*n* = 86). A total of 21,359 (4.2%) reports were finally included in this analysis.

**Results:**

More than half of the ADR reports were for children aged < 10 years. Approximately 30% of ADR reports had multiple suspected drugs, which did not differ by age. The percentages of fatal outcomes of ADRs among patients aged < 10 and 10–19 years were 4.7 and 3.9%, respectively. The most frequently reported drug, reaction, and drug-reaction pair were *oseltamivir*, *abnormal behavior*, and *oseltamivir and abnormal behavior*, respectively.

**Conclusion:**

We clarified the characteristics of ADR reports for Japanese children by using the JADER. ADR report databases, especially those for pediatric patients, are valuable pharmacovigilance tools in Japan and other countries. Therefore, a proper understanding of the characteristics of the ADR reports in the JADER is important. Additionally, potential signals for ADRs in pediatric patients should be monitored continuously and carefully.

## Background

Spontaneous reporting systems for adverse drug reactions (ADRs) are essential for post-marketing drug safety surveillance [1]. Such systems have been widely used for many drug safety studies. Because nationally compiled data, especially pediatric patient data, may be limited, the Global Research in Pediatrics-Network of Excellence (GRiP) network aims to facilitate the development and safe use of medicine in children and is valuable for examining drug safety [2]. The GRiP project describes the characteristics of individual case safety reports (ICSRs) as reported in a spontaneous reporting database operated by Food and Drug Administration in the United States [1]. Although, in general, a spontaneous ADR reporting database has some limitations such as a lack of denominator of users, an understanding of the structure and scope of the datasets and the respective strengths and limitations of such a database is essential for correct use and interpretation. An understanding of the characteristics of a database is the first and important step for evaluating and developing new methodologies for pharmacovigilance or drug safety [1]. Several retrospective studies of database for ADR reports have clarified their characteristics and availability for use as a database of drug safety surveillance among children in other countries, including the United States [3], Sweden [4], the United Kingdom [5], France [6], Malaysia [7], Spain [8], and Nigeria [9].

The regulatory authority in Japan began collecting ADR reports after the enactment of a law in 1961. Information on serious ADRs from individual cases and study reports from industries, direct voluntary reports from medical institutions, study results from treatment outcome studies, and post-marketing clinical trials has been accumulated since the enactment of the law. Post-2004 ADR reports have been compiled in the Japanese Adverse Drug Event Report database (JADER), which includes some items from ICSRs, such as patient demographic information, drug information, adverse events, and primary illness. This information became available for free download to anyone from the Pharmaceutical and Medical Devices Agency (PMDA) website since 2012 (*https://www.info.pmda.go.jp/fukusayoudb/CsvDownload.jsp*). This pharmacovigilance database provides a general picture of ADRs and suggests the relative plausibility using quantitative signal detection methodologies. However, there are no studies investigating the characteristics and utility of the JADER as a resource for drug safety surveillance in pediatric patients. Hence, in this study, we studied ADR reporting for pediatric patients in the JADER with an aim to elucidate the characteristics of the ADR reports therein in pediatric patients.

## Methods

We used spontaneous ADR reports included in the JADER since April 1, 2004, to December 31, 2017, which was downloaded in April 2018. The ADR reports are checked and evaluated whether the ADR report is serious or not before being registered in the JADER by the PMDA, and the JADER in principle comprises serious ADR reports selected by the PMDA. A single ADR report often includes multiple ADRs, which can include non-serious events such as pyrexia and rash. The PMDA recommend companies and healthcare professionals to report ADRs through a system called the Drugs and Medical Devices Safety Information Reporting System, even if the causal relationship between medication use and ADR was unclear. As for patients, the Direct Patient Reporting System for ADR, in which patients and consumers can report ADRs directly to the PMDA, was tentatively started from 2012 as a pilot program and a full-scale operation of the system was started on March 26, 2019. However, the JADER has not included the reports from this system yet. The JADER consists of four tables: (1) patient demographic information (2) drug information (3) adverse events, and (4) primary disease. We extracted spontaneous reports from companies and healthcare facilities. Spontaneous reports were defined as ADR reports derived from unsolicited sources in the International Conference on Harmonization of Technical Requirements for Registration of Pharmaceuticals for Human Use guideline E2B, which included direct reports from healthcare facilities or companies, ADR reports from abstracts, literature, Internet, etc. Because a different system exists for the reporting of adverse reactions due to vaccines, vaccine reports were excluded. The adverse reaction and primary disease fields in the JADER are described by using the Medical Dictionary for Regulatory Activities/Japanese version (MedDRA®/J) and were coded as preferred terms (PTs). We used MedDRA®/J Version 21.0 in the present study. The information included patient details (age and sex), type of report sender, reporters, suspected drugs, outcomes from ADR reports, and ADRs coded according to PTs. Age, sex, type of report sender (company or healthcare facility), reporters (doctor, pharmacist, healthcare professional, consumer, or lawyer), number of suspected drugs per ADR report, outcomes from ADR reports (cured, recovering, did not recover, recovering with sequelae, death, or unexplained) were collected. As for suspected drugs, we collected both International Nonproprietary Name (INN) and brand name and used INN to treat drugs with the same ingredients as the same drugs for analysis. Since the JADER only included age information as a categorical variable, we extracted ADR reports for children aged < 10 and 10–19 years. The 10 most frequently reported drugs, reactions, and drug-reaction pairs were determined according to age (< 10 years and 10–19 years). Time trends for the number of reports and the frequently reported drug, reaction, and drug-reaction pair were also determined. Adverse events were considered serious when they resulted in death, were life threatening, required hospitalization or prolongation of existing hospitalization, resulted in persistent or significant disability or incapacity, were congenital abnormalities or birth defects or were any other medically significant events.

## Results

### Characteristics of the reports in the JADER

**Fig. 1:**
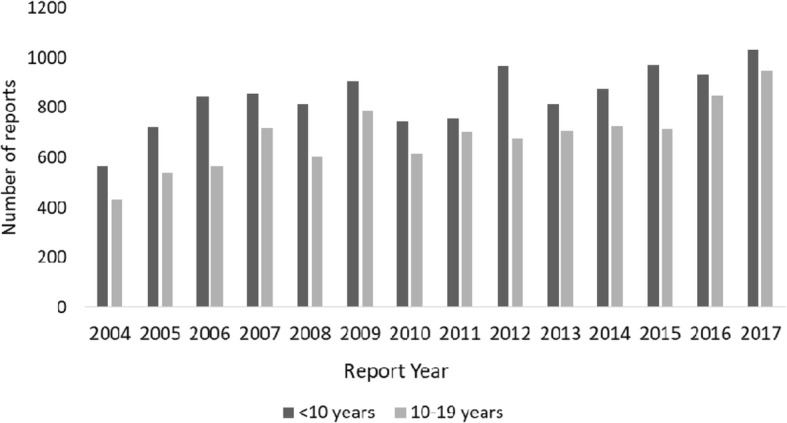
Annual ADR reports pertaining to children in Japan for 2004–2017 according to age group. ADR: adverse drug reaction

A total of 504,407 ADR reports from April 2004 to December 2017 were downloaded from the JADER in April 2018. Of these, the number of spontaneous reports was 386,400 (76.6%), in which 37,534 (7.4%) were unknown age reports. After extraction of 27,800 ADR reports for children aged < 10 and 10–19 years, we excepted for ADR reports associated with a vaccine (*n* = 6355) and no-suspected drug reports (*n* = 86). A total of 21,359 (4.2%) reports were finally included in this analysis. More than half of the ADR reports pertained to children aged < 10 years (Table 1). In the ADR notifications, the distribution of patients by sex was 53.5% boy and 40.5% girl for patients aged < 10 years and 51.3% boy and 46.5% girl for patients aged 10–19 years. Regardless of age, most of the reports in the JADER were sent by companies and > 70% were sent by doctors. Figure 1 shows the steadily increasing trend in the number of ADR reports. Approximately 30% of ADR reports had multiple suspected drugs, which did not differ by age (Table 1). For patients aged < 10 years, there were 11,786 ADR reports in total, of which 552 (4.7%) were fatal ADR reports with death reported as an outcome. For patients aged 10–19 years, there were 9573 ADR reports in total, of which 369 (3.9%) were fatal ADR reports with death as an outcome. The proportion of fatal ADR reports was higher when ADR reports had multiple suspected drugs (Table 1).

**Table 1:** Characteristics of ADR reports according to age group

	Age group	
	< 10 years *n* = 11,786 (55.2%)	10–19 years *n* = 9573 (44.8%)
Sex		
Boy, n (%)	6305 (53.5)	4910 (51.3)
Girl, n (%)	4777 (40.5)	4449 (46.5)
Unexplained, n (%)	704 (6.0)	214 (2.2)
Report source		
Company, n (%)	11,652 (98.9)	9430 (98.5)
Healthcare facility, n (%)	134 (1.1)	143 (1.5)
Reporter		
Doctor, n (%)	10,002 (78.4)	7901 (74.8)
Pharmacist, n (%)	1218 (9.5)	1208 (11.4)
Healthcare professional, n (%)	430 (3.4)	480 (4.5)
Consumer, n (%)	449 (3.5)	501 (4.7)
Lawyer, n (%)	2 (0.0)	0 (0.0)
Unexplained, n (%)	656 (5.1)	472 (4.5)
Total, n (%)	12,757 (100)	10,562 (100)
Number of suspected drugs per ADR report		
1, n (%)	8248 (70.0)	6679 (69.8)
2, n (%)	1824 (15.5)	1449 (15.1)
3, n (%)	798 (6.7)	634 (6.6)
4, n (%)	394 (3.3)	322 (3.4)
5, n (%)	234 (2.0)	165 (1.7)
6, n (%)	100 (0.8)	131 (1.4)
7, n (%)	71 (0.6)	68 (0.7)
8, n (%)	52 (0.4)	33 (0.3)
9, n (%)	24 (0.2)	31 (0.3)
≥ 10, n (%)	41 (0.3)	61 (0.6)
Number of suspected drugs per fatal ADR report (n)		
1, n (%)	328 (4.0)	215 (3.2)
2, n (%)	93 (5.1)	61 (4.2)
3, n (%)	57 (7.1)	38 (6.0)
4, n (%)	28 (7.1)	16 (5.0)
≥ 5, n (%)	46 (8.8)	39 (8.0)
Total, n (%)	552 (4.7)	369 (3.9)

Abbreviation: ADR: adverse drug reaction

### Outcomes associated with ADR reports

For patients aged < 10 years, in the 11,786 reports, a total of 18,309 ADRs were reported. The percentages of patients who were cured, recovering, and recovering with sequelae were 43.1% (*n* = 7898), 23.4% (*n* = 4288), and 1.8% (*n* = 338), respectively; 5.4% (*n* = 993) of the patients did not recover. The percentage of fatal outcomes was 4.4% (*n* = 803). For patients aged 10–19 years, in the 9573 reports, a total of 15,419 ADRs were reported. The percentages of patients who were cured, recovering, and recovering with sequelae were 44.1% (*n* = 6805), 22.6% (*n* = 3492), and 1.1% (*n* = 162), respectively; 4.4% (*n* = 684) of the patients did not recover. The percentage of fatal outcomes was 3.3% (*n* = 512).

### Frequently reported drugs

**Fig. 2:**
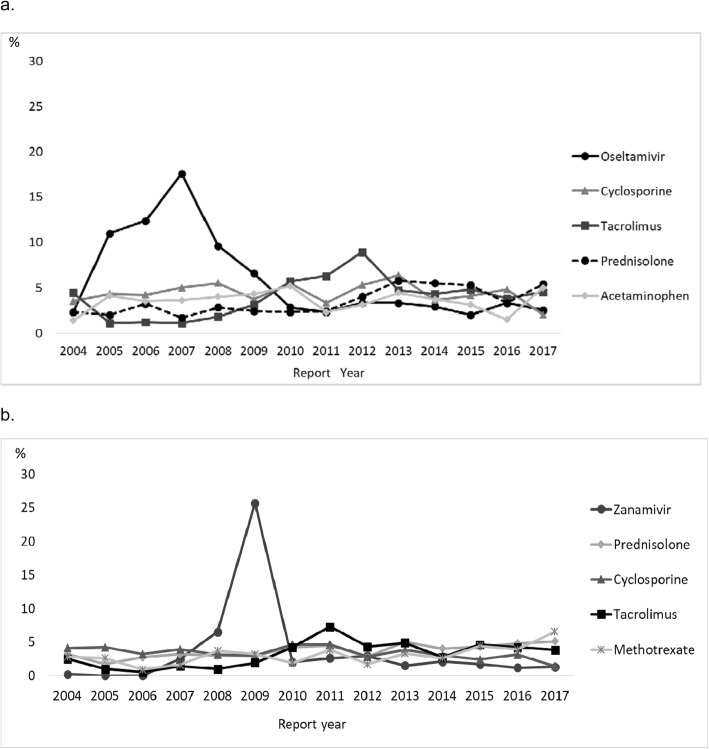
Time trend for the five most frequently reported drugs according to age group. **a** children aged < 10 years; **b** children aged 10–19 years

The most frequently reported drugs in ADR reports for patients aged < 10 and 10–19 years were oseltamivir (2.8%) and zanamivir (2.7%), respectively. There were many ADR reports associated with immunosuppressants such as tacrolimus, cyclosporine, and prednisolone, which did not differ according to age. (Table 2). There were many ADR reports for oseltamivir from 2004 to 2008, especially in 2007 for patients aged < 10 years. In 2009, there were many ADR reports for zanamivir for patients aged 10–19 years (Fig. 2). Among 1128 and 764 reported drugs of 552 and 369 fatal ADR reports for patients aged < 10 and 10–19 years, the most frequently reported drugs were etoposide (3.6%) and tacrolimus (5.1%), respectively.

**Table 2:** Ten most frequently reported drugs according to age group

a. < 10 years (*n* = 19,829)	
Oseltamivir, n (%)	540 (2.8)
Cyclosporine, n (%)	413 (2.1)
Tacrolimus, n (%)	387 (2.0)
Prednisolone, n (%)	343 (1.8)
Acetaminophen, n (%)	341 (1.8)
Sodium valproate, n (%)	313 (1.6)
Carbamazepine, n (%)	304 (1.6)
Methotrexate, n (%)	258 (1.3)
Cefditoren pivoxil, n (%)	257 (1.3)
Ceftriaxone sodium, n (%)	249 (1.3)
b. 10–19 years (n = 16,552)	
Zanamivir, n (%)	440 (2.7)
Prednisolone, n (%)	439 (2.7)
Cyclosporine, n (%)	384 (2.3)
Tacrolimus, n (%)	375 (2.3)
Methotrexate, n (%)	371 (2.2)
Carbamazepine, n (%)	370 (2.2)
Acetaminophen, n (%)	303 (1.8)
Oseltamivir, n (%)	295 (1.8)
L-Asparaginase, n (%)	244 (1.5)
Cyclophosphamide, n (%)	236 (1.4)

### Frequently reported reactions

**Fig. 3:**
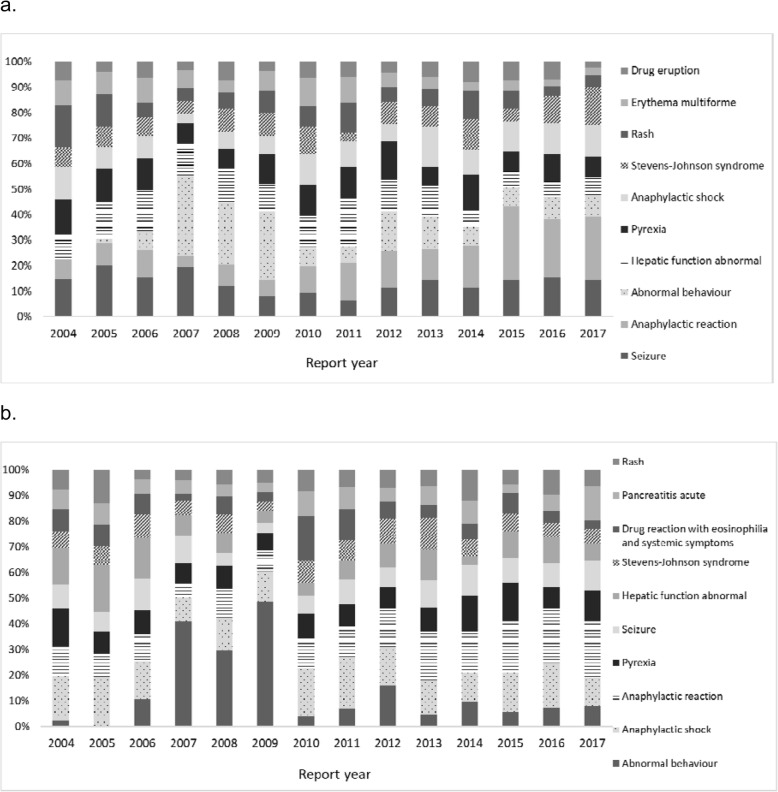
Time trend for the 10 most frequently reported adverse reactions according to age group. **a** children aged < 10 years; **b** children aged 10–19 years

For patients aged < 10 and 10–19 years, the most frequently reported reactions were seizure (2.2%) and abnormal behavior (2.8%), respectively (Table 3). The time trend for frequently reported reactions was abnormal behavior from 2007 to 2009, and it did not differ by age (Fig. 3). Among 1095 and 768 reported drugs of 552 and 369 fatal ADR reports for patients aged < 10 and 10–19 years, the most frequently reported reactions were" death" (3.0%) and sepsis (3.4%), respectively.

**Table 3:** Ten most frequently reported reactions according to age group

a. < 10 years (*n* = 18,022)	
Seizure, n (%)	390 (2.2)
Anaphylactic reaction, n (%)	374 (2.1)
Abnormal behavior, n (%)	374 (2.1)
Hepatic function abnormal, n (%)	336 (1.9)
Pyrexia, n (%)	319 (1.8)
Anaphylactic shock, n (%)	271 (1.5)
Stevens-Johnson syndrome, n (%)	246 (1.4)
Rash, n (%)	229 (1.3)
Erythema multiforme, n (%)	195 (1.1)
Drug eruption, n (%)	156 (0.9)
b. 10–19 years (*n* = 15,157)	
Abnormal behavior, n (%)	419 (2.8)
Anaphylactic shock, n (%)	353 (2.3)
Anaphylactic reaction, n (%)	333 (2.2)
Pyrexia, n (%)	236 (1.6)
Seizure, n (%)	223 (1.5)
Hepatic function abnormal, n (%)	220 (1.5)
Stevens-Johnson syndrome, n (%)	175 (1.2)
Drug reaction with eosinophilia and systemic symptoms, n (%)	171 (1.1)
Pancreatitis acute, n (%)	170 (1.1)
Rash, n (%)	169 (1.1)

Note: The terms are as described in Japanese version 21.0 of MedDRA®

### Frequently reported drug-reaction pairs

**Fig. 4:**
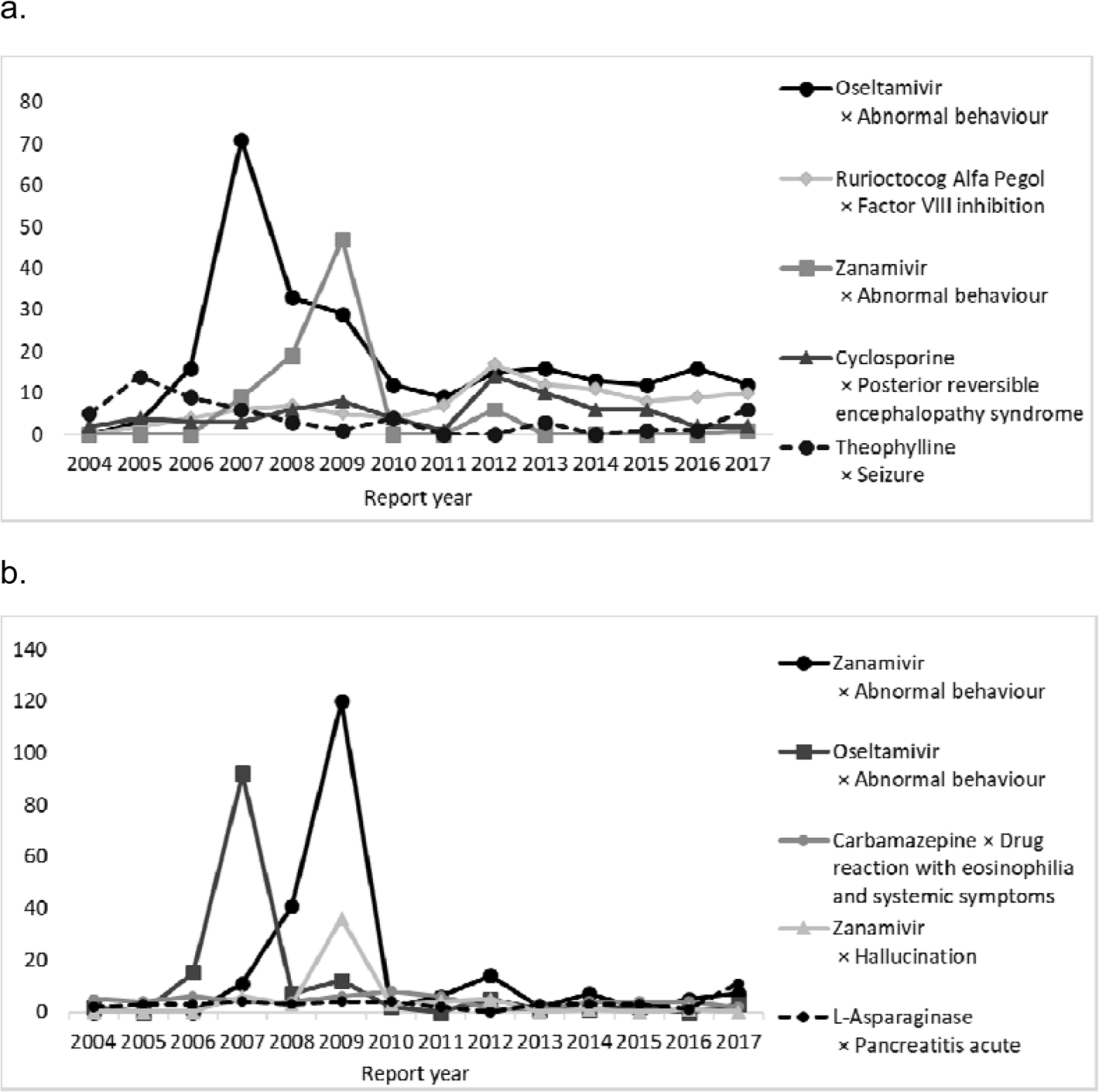
Time trend for the five most frequently reported drug-reaction pairs according to age group. **a** children aged < 10 years; **b** children aged 10–19 years

The most frequently reported drug-reaction pairs were “oseltamivir and abnormal behavior” (0.8%) and “zanamivir and abnormal behavior” (0.8%) in patients aged < 10 and 10–19 years, respectively (Table 4). The time trends for frequently reported drug-reaction pairs were “oseltamivir and abnormal behavior” in 2007 and “zanamivir and abnormal behavior” in 2009, which did not differ by age (Fig. 4). Among 2363 and 1852 reported drug-reaction pairs of 552 and 369 fatal ADR reports for patients aged < 10 and 10–19 years, the most frequently reported drug-reaction pairs were “etoposide and acute respiratory distress syndrome” (0.3%) and “bortezomib and neutropenia” (0.4%), respectively.

**Table 4:** Ten most frequent drug-reaction pairs according to age group

Generic name	Reaction	n (%)
a. < 10 years		
Oseltamivir	Abnormal behavior	257 (0.8)
Rurioctocog alfa pegol (Genetical recombination)	Factor VIII inhibition	102 (0.3)
Zanamivir	Abnormal behavior	82 (0.3)
Cyclosporine	Posterior reversible encephalopathy syndrome	71 (0.2)
Theophylline	Seizure	53 (0.2)
Acetaminophen	Toxic epidermal necrolysis	52 (0.2)
Theophylline	Encephalopathy	50 (0.2)
Oseltamivir	Seizure	48 (0.2)
Acetaminophen	Stevens-Johnson syndrome	44 (0.1)
Amoxicillin	Erythema multiforme	44 (0.1)
b. 10–19 years		
Zanamivir	Abnormal behavior	216 (0.8)
Oseltamivir	Abnormal behavior	142 (0.5)
Carbamazepine	Drug reaction with eosinophilia and systemic symptoms	59 (0.2)
Zanamivir	Hallucination	59 (0.2)
L-Asparaginase	Pancreatitis acute	46 (0.2)
Cyclosporine	Nephropathy toxic	42 (0.2)
Irradiated platelet concentrate, leukocytes reduced	Anaphylactic reaction	38 (0.1)
Lamotrigine	Rash	38 (0.1)
Levetiracetam	Epilepsy	37 (0.1)
Vincristine	Neutropenia	37 (0.1)

## Discussion

In this study, the number of ADR reports from reporters other than companies, especially pharmacists are low. Although most Japanese hospital pharmacists sufficiently understood the spontaneous ADR reporting system, they also had some barriers to report the ADR such as what kind of ADR to be reported [10]. Additionally, companies are required strictly to report all ADRs within the reporting deadline, differently from healthcare facilities. Therefore, compared to the healthcare facility, the number of ADR reports from the company might be relatively high.

In Japan, 3.3–4.4% of pediatric ADR reports during the study period were related to fatal cases, which was higher than the corresponding percentage in other countries (0.37% in the UK, 0.24% in Malaysia, and 0.49% in Spain) [7, 8, 11]. One of the reasons for the higher percentage of fatal cases was that the JADER is a spontaneous ADR database that in principle comprises serious ADR reports selected by the PMDA and databases in other countries included non-serious ADRs. Therefore, the percentage of fatal cases may reflect differences in the use of medicines and attitudes toward reporting in different countries [12].

Fatal ADR reports are the cases where outcomes are described as death and tend to be reported more positively because of their importance and difficulty in understanding. Our study found that the percentage of fatal ADR reports was higher when ADR reports had multiple suspected drugs. Although polypharmacy might reflect a severe disease that requires the use of multiple drugs, a previous assessment of the severity of the reported ADRs found that multiple drug exposure might more often lead to serious ADR reports compared to single drug use [13]. Another study found that the use of more than four drugs simultaneously positively correlated with ADR occurrence [14]. Polypharmacy increases the chance of drug-drug interactions and the possibility of ADR occurrence [15, 16]. Because our finding was based on the examination of spontaneous reports, we simply observed reporting tendency. However, considering previous findings in addition to our own, we may pay particular attention to ADRs for children who are prescribed two or more drugs to minimize the risk of serious ADRs.

This study showed that there are many ADR reports associated with immunosuppressants. Because the JADER is a database comprising serious ADR reports, it might contain a lot of information about drugs that are likely to cause serious ADRs. As for the most frequently reported drugs in Table 2, the number of ADR reports regarding oseltamivir might be increased by the Dear Healthcare Professional Letters. All drugs in the list, safety information regarding the revision of the precautions of package inserts of drugs have been provided in Pharmaceuticals and Medical Devices Safety Information published by Ministry of Health, Labour and Welfare or Drug Safety Update published by the Federation of Pharmaceutical Manufacturers’ Associations of Japan. This information might have boosted the number of ADR reports. Therefore, the list of drugs in Table 2 seemed not to be related to drug use.

The characteristics of ADR reports varied considerably by the pediatric patient age in previous reports [7]. The potential risk of serious adverse events varies with age and the variability in ADRs by pediatric patient age also differs depending on whether children can complain of side effects. In other words, objective reactions may be reported more often by younger children than by older children, and subjective reactions may be reported more often by older children than by younger children. Therefore, information on age is essential in discussions about ADRs, especially in pediatric patients. However, the present study could not obtain age-related information as a continuous variable and it was used as a categorical variable, such as ‘< 10 years’ and ‘10–19 years’, and it was the weakest attribute related to the JADER. In this study, objective reactions were mainly reported. Pediatric ADR reports, therefore, need to be considered with a more detailed age classification. Age information should have been reported as a continuous variable in original ADR reports; however, the JADER only includes age information as a categorical variable because of privacy considerations. To increase the availability and value of the JADER, age information as a continuous variable should be disclosed, especially in pediatric ADR reports.

Many abnormal behaviors related to oseltamivir administration were reported in 2007 and many abnormal behaviors related to zanamivir were reported in 2009. Abnormal behaviors related to oseltamivir created concern, and the Dear Healthcare Professional Letters about the abnormal behaviors related to oseltamivir were published by a Japanese regulatory agency on November 27, 2007. In addition, the use of zanamivir, a similar drug to oseltamivir, increased with the advent of the oseltamivir-resistant virus in 2008–2009. In early post-marketing phase vigilance (EPPV), a unique system of post-marketing surveillance started in October 2001 in Japan, medical representatives regularly visit medical institutions during the first 6 months of marketing to collect ADRs, so a positive association between the EPPV period and the number of ADRs reported has been suggested [17, 18]. However, EPPV did not have a positive impact on the increase in the number of ADR reports regarding abnormal behavior related to oseltamivir in 2007 because EPPV for oseltamivir was conducted in 2002 in Japan. ADR reports on “oseltamivir and abnormal behavior” and “zanamivir and abnormal behavior” were thought to have become frequent just after the publication of the letters, although the causal relationship between oseltamivir and abnormal behavior has not been clarified. However, because the percentage of reports on “anti-influenza virus drugs and abnormal behavior” was not so much (1.1% under 10 years old and 1.4% among children aged 10–19 years), those pairs might not influence on detecting the other signals. In Spain, after the publication of warnings on the use of antidepressants and treatment of attention deficit disorder and hyperactivity linked to the risk of cardiovascular and cerebrovascular disorders in pediatric patients by regulatory agencies, the number of ADR reports regarding cardiovascular and cerebrovascular disorders following the use of antidepressants and treatment of attention deficit disorder and hyperactivity increased [8]. The number of reports of toxic epidermal necrolysis (TEN) and Stevens-Johnson syndrome (SJS) associated with acetaminophen was also very high. This result might be explained by some reasons as follow; acetaminophen is often used for children, and initiation of acetaminophen treatment occurs in response to fever or ear, nose and throat pain, which might be often the prodromal symptoms of SJS/TEN and that of an infectious disease such as mycoplasma infection or a viral Infections such as influenza accountable for SJS/TEN [19–21].

ADR monitoring based on spontaneous reports in children is an important safety-monitoring activity compared to that in adults because there are few foundations for evaluating the safety of drugs in children. However, the actual causal relationship needs to be continuously verified separately even if many spontaneous reports have observed and regulatory authorities have issued warnings. It should be recognized that the JADER, a spontaneous report database in Japan, also includes such reports that are not clear the causal relationship.

The present study has several limitations. First, the JADER is a passive system, marked by multiple limitations, such as reporting of temporal association, unconfirmed diagnoses, a lack of denominator of users, and unbiased comparison grope [22]. Because of these limitations, it is usually not possible to establish causality between drugs and adverse reactions from JADER reports. Second, it was not possible to analyze the situation according to WHO age group classification such as children aged 5–17 years because the JADER only included age information as a categorical variable such as children aged < 10 and 10–19 years. Nomura et al. have already compared Japanese ADR reports between the FAERS and the JADER [23]. Although the FAERS included non-US data received by drug companies worldwide and it was possible to select Japanese reports with detailed information for age, they clarified that the FAERS and the JADER had different properties. Therefore, in our study, we clarified the characteristics of ADR reports for Japanese children by using the JADER. Third, in the JADER, detailed information on the source of spontaneous ADR reports was not revealed. Therefore, there remains the possibility of duplicated reports, whereby one case might be reported multiple times. This possibility cannot be completely excluded because there are no identifiers for the same case. The identification and elimination of duplicates from an analysis are advantageous and important for the correct interpretation of the data. In future studies, we will evaluate the ability of the JADER for signal detection based on the characteristics of the JADER clarified in this study.

## Conclusion

We clarified the characteristics of ADR reports for Japanese children by using the JADER. ADR report databases, especially those for pediatric patients, are valuable pharmacovigilance tools in Japan and other countries. Therefore, a proper understanding of the characteristics of the ADR reports in the JADER is important and several limitations such as age group and duplicated reports need to be improved. Additionally, potential signals for ADRs in pediatric patients should be monitored continuously and carefully.

## Data Availability

The datasets used and/or analyzed during the current study are available from the corresponding author on reasonable request.

## Abbreviations

ADR: adverse drug reaction
JADER: Japanese Adverse Drug Event Report database
GRiP: Global Research in Pediatrics-Network of Excellence
ICSRs: individual case safety reports
PMDA: Pharmaceutical and Medical Devices Agency
EPPV: early post-marketing phase vigilance
TEN: toxic epidermal necrolysis
SJS: Stevens-Johnson syndrome
